# Efficacy of Modified Talc Powder in Experimental Rat Model of Pleurodesis

**DOI:** 10.3390/biom16010104

**Published:** 2026-01-07

**Authors:** Murat Kilic, Onural Ozhan, Azibe Yildiz, Süleyman Koytepe, Mustafa Akyuz, Yusuf Turkoz, Nurcan Gokturk, Merve Biyikli, Rumeysa Sonmez, Idil Karaca Acari, Hakan Parlakpinar

**Affiliations:** 1 Department of Thoracic Surgery, Faculty of Medicine, Inonu University, 44280 Malatya, Turkey; merve.biyikli@inonu.edu.tr; 2 Department of Medical Pharmacology, Faculty of Medicine, Inonu University, 44280 Malatya, Turkey; onural.ozhan@inonu.edu.tr (O.O.); 38244201001@ogr.inonu.edu.tr (R.S.);; 3 Department of Histology and Embryology, Faculty of Medicine, Inonu University, 44280 Malatya, Turkey; azibe.yildiz@inonu.edu.tr (A.Y.); mustafa.akyuz@inonu.edu.tr (M.A.); 4 Department of Chemistry, Faculty of Science, Inonu University, 44280 Malatya, Turkey; suleyman.koytepe@inonu.edu.tr; 5 Department of Medical Biochemistry, Faculty of Medicine, Inonu University, 44280 Malatya, Turkey; yusuf.turkoz@inonu.edu.tr (Y.T.); gokturk.44@hotmail.com (N.G.); 6 Department of Bioengineering, Faculty of Engineering and Natural Sciences, Malatya Turgut Ozal University, 44330 Malatya, Turkey

**Keywords:** pleurodesis, modified talc, pleural cavity, lung, rat model

## Abstract

**Background:** Pleurodesis is a treatment method that aims to create permanent adhesion between the pleural layers to prevent recurrent fluid or air accumulation in the pleural cavity. Talc, one of the most commonly preferred agents in this procedure, is widely used in clinical practice. In this study, a new talc formulation with a modified surface to impart antibacterial and analgesic properties was experimentally evaluated for the first time. The main objective of the study was to comparatively assess the inflammatory and fibrotic responses following standard talc and modified talc applications. **Methods:** Thirty-six 12-week-old female Wistar albino rats were simply randomly divided into three different groups: control (*n* = 12), standard talc (*n* = 12), and modified talc (*n* = 12). Under anesthesia, 1 mL of physiological saline containing 17 mg of talc was injected intrapleurally into the right hemithorax. The presence of pneumothorax after the procedure was assessed by chest radiography. After a 12-day follow-up period, the animals were euthanized. Bronchoalveolar lavage (BAL) fluid samples, blood samples, and lung and pleural tissue samples were collected for biochemical, histopathological, and immunohistochemical analyses. **Results:** Modified talc application resulted in a significant increase in both visceral and parietal pleural thickness (*p* < 0.05). Granulation tissue formation and collagen deposition were significantly higher in the modified talc group. In addition, TGF-β expression and CD68-positive macrophage count increased significantly in the modified talc group (*p* < 0.05). Inflammatory changes in the lung parenchyma were limited and not statistically significant. **Conclusions:** The modified talc formulation enriched with lidocaine and antibacterial agents produced a stronger inflammatory and fibrotic response compared to standard talc. These findings indicate that modified talc may increase the effectiveness of pleurodesis. Furthermore, the absence of significant lung parenchymal damage suggests that this treatment is locally effective and feasible. However, further long-term and advanced studies are needed to translate these results into clinical use.

## 1. Introduction

Recurrent pleural effusion and pneumothorax are conditions that can cause serious symptoms and significantly impair a patient’s quality of life. One of the methods frequently used in the treatment of this condition is pleurodesis, which aims to trigger pleural inflammation, create adhesions between the visceral and parietal pleural layers, and permanently eliminate the pleural cavity [1,2]. Although various agents such as doxycycline, bleomycin, and silver nitrate have been tried for pleurodesis, these agents have not gained the characteristic of being an ideal pleurodesis agent due to either insufficient pleurodesis efficacy or reasons such as significant pain and toxicity. In addition, the vast majority of existing agents do not have analgesic and antimicrobial properties. The pain that occurs after pleurodesis is a significant clinical problem that reduces patient comfort and leads to the need for additional analgesics. Therefore, the search for an effective and ideal pleurodesis agent continues. Today, talc, which is used as a pleurodesis agent, is one of the most preferred sclerosing agents due to its easy availability, low cost, high efficacy, and ease of application. However, despite the frequent use of talc pleurodesis, side effects such as the risk of systemic inflammatory response (which varies depending on particle size), severe pulmonary complications, acute respiratory distress syndrome (ARDS), and changes in circulating volume make the reliability of the method controversial. In particular, talc with a small particle size (<5 µm) causes a more widespread and potent systemic inflammatory response, leading to a marked increase in the levels of cytokines such as interleukin-8 (IL-8), vascular endothelial growth factor (VEGF), and transforming growth factor beta (TGF)-β [3]. This cytokine-mediated inflammation extends beyond the pleura into the circulation, resulting in systemic manifestations such as fever, elevated CRP, and leukocytosis [4]. Neutrophil infiltration, primarily mediated by IL-8, occurs rapidly, whereas fibrosis mediated by TGF-β develops over a longer period. Such systemic responses complicate the assessment of local inflammatory changes, thereby limiting both safety and efficacy evaluations [5,6]. Moreover, pleuritic pain and patient intolerance during talc application may further compromise treatment [7]. Antibiotic treatment administered prior to pleurodesis and prolonged drainage duration were risk factors for empyema following pleurodesis [8].

For these reasons, research efforts have focused on modifying talc-based agents to enhance efficacy while reducing adverse effects. In this context, a novel talc formulation has been developed: the talc surface was modified with 3-vinylpropyltriethoxysilane to confer antibacterial properties, and lidocaine was incorporated to provide an analgesic effect. The rationale underpinning this project was to enhance the direct application of talc and to generate a functional biomedical material. Specifically, the talc structure was first modified to contain antibacterial groups, after which lidocaine was loaded to confer both analgesic and potential anti-inflammatory properties. However, the impact of this modified talc formulation on pleurodesis remains to be investigated, thereby offering an innovative approach.

Nevertheless, the repercussions of this modified talc formulation on pleurodesis have not yet been examined; thus, the present study proffers an innovative approach in this regard.

## 2. Materials and Methods

### 2.1. Drugs and Chemicals

In this study, modified talc with physicochemical properties reported in a previous study was used [9]. This novel formulation, which has not yet been evaluated in experimental studies, was obtained by subjecting the standard talc structure to surface modification to impart antibacterial properties. During the modification process, a coupling agent, triethoxyvinylsilane (TEVS), was incorporated into appropriately sized talc particles, thereby generating functional groups on the particle surface. TEVS was supplied by Sigma-Aldrich Chem. Co. (St. Louis, MO, USA). The modified surface was designed according to the ‘kill and release’ principle. That is, it was engineered to inactivate bacteria upon contact and subsequently release the bacterial residues from the surface. Furthermore, lidocaine was incorporated into the talc formulation as a local anesthetic component, with the objective of minimizing pain during intrapleural administration.

### 2.2. Animals and Study Design

The protocol for this study was approved by the İnonu University Local Animal Experimentation Ethics Committee (Date 28 June 2024 No: 2024/12-9). Twelve-week-old female Wistar albino rats (*n* = 36), weighing 200–300 g, were used. The animals were obtained from the İnönü University Laboratory Animal Production and Research Center. During the experiment, they were housed in appropriately sized polycarbonate cages at 21 ± 2 °C and provided with ad libitum access to standard rodent chow and water. All animal care was performed under the supervision of a veterinarian in accordance with the Turkish Animal Protection Law. The National Institutes of Health’s Guide for the Care and Use of Laboratory Animals and the ARRIVE guidelines (Animals in Research: Reporting In Vivo Experiments) were followed throughout all experimental procedures [10].

**Figure 1:**
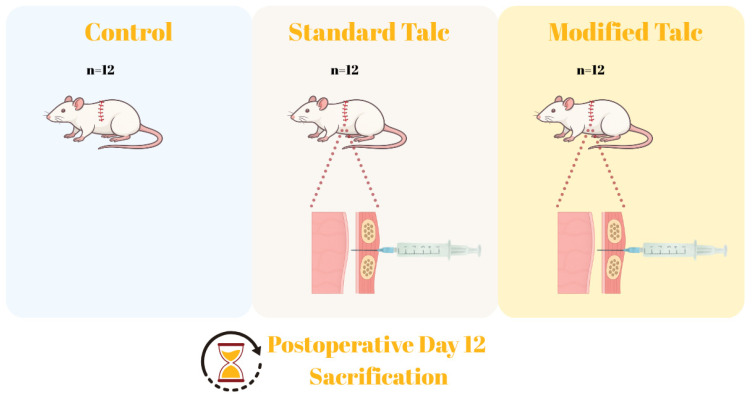
Experimental groups: Rats were divided into the standard talc group, the modified talc group, and the control group.

Rats were randomly assigned to three groups with equal numbers of animals per group (see Figure 1):

Group 1—Standard talc (*n* = 12): 17 mg of standard talc powder suspended in 1 mL of isotonic solution was administered intrapleurally.

Group 2—Modified talc (*n* = 12): 17 mg of modified talc suspended in 1 mL of isotonic solution was administered intrapleurally.

Group 3—Control (*n* = 12): 1 mL of physiological saline was administered intrapleurally.

### 2.3. Surgical Procedure and Sample Collection

General anesthesia was achieved using a combination of ketamine (100 mg/kg) and xylazine (8 mg/kg) administered intraperitoneally. Following anesthesia, the right hemithorax of each rat was shaved and disinfected with 1% povidone–iodine solution. The surgical site was illuminated and magnified using a lighted magnifying loop. Under sterile conditions, a 5 mm skin incision was made at the level of the 5th intercostal space along the right posterior axillary line. The chest wall muscles were dissected, and the superficial fibers of the intercostal muscles were carefully cut with microsurgical scissors, preserving the integrity of the parietal pleura.

**Figure 2:**
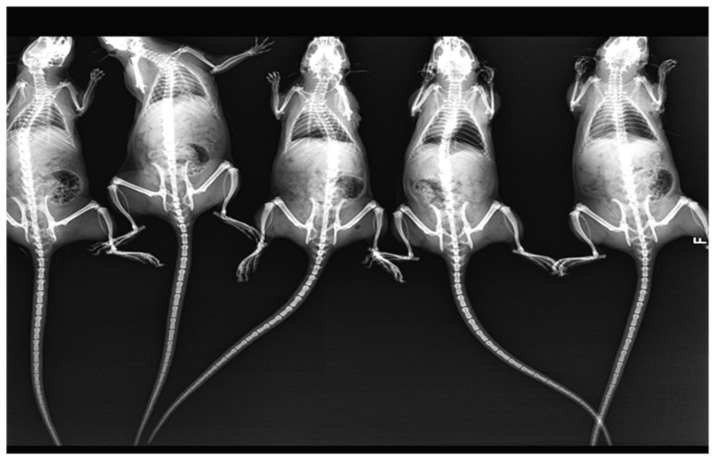
Chest radiography of rats after pleurisy procedure.

While 20-gauge polytetrafluoroethylene catheters are frequently used for intrapleural applications in the literature, a round-tipped, blunt-ended gavage cannula was used in this study. The gavage cannula offered several advantages:

1. The blunt tip allowed safe and rapid access to the pleural space.
2. Any free air formed after the procedure could be safely aspirated with negative pressure while the cannula remained in the thorax, reducing the risk of lung injury and pneumothorax. This was confirmed by postoperative chest radiographs (see Figure 2).

After the procedure, the cannula was slightly withdrawn, and the air in the pleural cavity was aspirated using negative pressure. Prolene 3/0 sutures placed around the cannula site were tied securely, the cannula was removed, and the incision was closed. Suture ends were kept short to prevent self-trauma. Postoperatively, animals were mobilized by turning them into right and left lateral positions to ensure even distribution of talc powder within the pleural cavity. During the surgical procedure, two rats from each of the standard talc and modified talc groups died prematurely. In the control group, one rat was lost due to an inadequate response to the initial anesthetic dose, and another died within 24 h post-surgery. All remaining animals survived until day 12, with no further losses. On the twelfth day, all rats were euthanized by exsanguination under appropriate anesthesia. Bronchoalveolar lavage (BAL) samples were obtained from each animal. Blood samples were collected via thoracotomy. The right hemithorax was removed en bloc, and lung and chest wall tissues were fixed in 10% neutral formalin and sent for histopathological examination.

- *Pain Assessment*

In this experimental study, no formal postoperative pain scoring system was applied. All intrapleural procedures were performed under general anesthesia using ketamine and xylazine, and no validated behavioral or facial pain assessment tools (such as the Rat Grimace Scale or standardized behavioral scoring systems) were prospectively included in the study design.

Lidocaine was incorporated into the modified talc formulation with the aim of reducing local nociceptive stimulation during intrapleural administration. However, due to the absence of predefined quantitative pain assessment criteria, postoperative pain levels could not be objectively measured or compared between the experimental groups.

### 2.4. Biochemical Analyses

Blood samples were collected from the study rats via the intracardiac route into gel separator tubes and centrifuged at 2000 rpm for 5 min to obtain serum. The concentrations of IL-6 and TNF-α in the serum were measured using commercial ELISA kits (BT-lab, Shanghai Korain Biotech Co., Ltd., Shanghai, China) according to the manufacturer’s instructions.

### 2.5. Histopathological and Immunohistochemical Analyses

Within the scope of the study, lung parenchyma, visceral pleura, and parietal pleura were fixed in 10% neutral formalin, dehydrated through graded alcohol and xylene series, and embedded in paraffin blocks. For histological evaluation, 4 µm thick sections were obtained from the paraffin-embedded tissue samples. The presence of granulation tissue in the visceral and parietal pleura was assessed using hematoxylin and eosin (H&E) staining. Twenty randomly selected fields per sample were evaluated under 400× magnification, with scoring as follows: 0 = absent, 1 = mild, 2 = moderate, 3 = intense [11]. Collagen density in the visceral and parietal pleura was graded using Masson’s trichrome staining in 20 randomly selected fields at 400× magnification: 0 = absent, 1 = sparse, 2 = moderately dense, 3 = very dense [12]. Additionally, the thickness of the visceral and parietal pleura was measured in 20 randomly selected areas [13]. The lung parenchyma was examined for emphysema and inflammatory cell infiltration in 10 randomly selected fields. Scores for each parameter were defined follows: 0 = normal tissue, 1 = damage <25% of the total area, 2 = damage 25–75%, 3 = damage >75% [14].

### 2.6. Immunohistochemical Analyses

CD68 was used to assess the presence of macrophages in lung tissue, while TGF-β was employed to evaluate pleural fibrosis. Tissue sections (4 µm thick) for immunohistochemistry were mounted on polylysine-coated slides. The sections were deparaffinized and rehydrated through xylene and a graded ethanol series. For antigen retrieval, sections were incubated in sodium citrate buffer (pH 6.0) and boiled in a pressure cooker for 20 min, followed by cooling at room temperature.

Subsequently, the sections were incubated with 0.3% hydrogen peroxide for 15 min to block endogenous peroxidase activity. To prevent nonspecific binding, a protein blocking reagent was applied for 5 min. Sections were then incubated with the primary antibodies (CD68 and TGF-β, 1:200; Santa Cruz Biotechnology, Inc., Heidelberg, Germany) at room temperature for 60 min.

Following primary antibody incubation, sections were treated with a biotinylated secondary antibody and then incubated with a streptavidin–peroxidase complex for 15 min. Immunoreactions were visualized using aminomethylcarbazole (AEC)(Thermo Fisher Scientific, Fremont, CA, USA) as a chromogenic substrate. Sections were counterstained with hematoxylin, mounted with coverslips using an appropriate sealing medium, and prepared for microscopic examination.

CD68-positive cells were counted in 20 randomly selected fields per section at 400× magnification by a blinded observer [15]. TGF-β immunoreactivity was assessed in 20 randomly selected fields per section according to prevalence and intensity. Extent was scored as follows: 0–25% = 0, 26–50% = 1, 51–75% = 3, 76–100% = 4 points; intensity was scored from 0 to 3 based on staining visibility. Total scores were calculated by multiplying prevalence and intensity scores [16]. All analyses were conducted by a blinded investigator using a Leica DFC280 light microscope and Leica Q Win Image Analysis System (Leica Micros Imaging Solutions Ltd.; Cambridge, UK).

### 2.7. Statistical Analysis

Statistical analyses were conducted using IBM SPSS Statistics (version 27.0; SPSS Inc., Chicago, IL, USA). Data distribution was assessed with the Shapiro–Wilk normality test. For normally distributed variables (serum IL-6 and TNF-α), parametric one-way ANOVA was applied, followed by Tukey’s post hoc test for multiple comparisons. Non-normally distributed data were analyzed using the non-parametric Kruskal–Wallis test, and significant results were further examined with Bonferroni-corrected Mann–Whitney U tests. A *p*-value of <0.05 was considered statistically significant. Data are presented as mean ± SEM for normally distributed variables and as median (minimum–maximum) for non-normally distributed variables.

During manuscript preparation, ChatGPT (GPT-4, OpenAI, San Francisco, CA, USA)was used only for language editing and clarity improvement (grammar, spelling, and sentence structure). No scientific content or data analysis was generated using AI tools.

## 3. Results

### 3.1. Pain Assessment Results

Since a standardized pain scoring system was not implemented in the experimental protocol, quantitative pain score comparisons between the control, standard talc, and modified talc groups could not be performed. No overt signs of severe distress or procedure-related complications requiring additional intervention were observed during the postoperative follow-up period in any of the groups.

### 3.2. Biochemical Results

**Figure 3:**
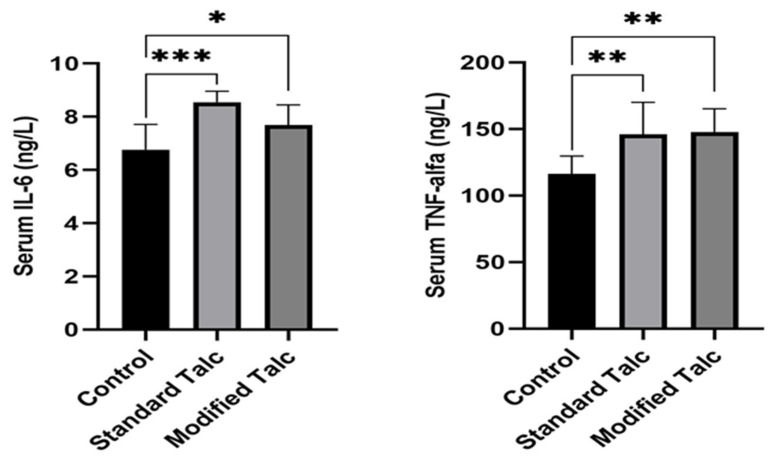
IL-6 and TNF-alpha levels in Standard Talc and Modified Talc groups. (*) means *p* < 0.05, (**) means *p* < 0.01, (***) means *p* < 0.001.

Serum IL-6 levels were 6.76 ± 0.317 ng/L in the control group, 8.54 ± 0.15 ng/L in the standard talc group, and 7.68 ± 0.23 ng/L in the modified talc group (mean ± SEM). Serum TNF-α levels were 116.20 ± 4.51 ng/L, 146.20 ± 9.07 ng/L, and 147.70 ± 5.54 ng/L in the control, standard talc, and modified talc groups, respectively. Serum IL-6 levels were significantly elevated in both the standard talc and modified talc groups compared to the control group (*p* < 0.0003 for standard talc, *p* < 0.0379 for modified talc), (Figure 3a). Standard talc increased serum IL-6 levels more markedly than modified talc (*p* < 0.0003). Serum TNF-α levels were also significantly higher in both talc-treated groups compared to the control (*p* < 0.0095 for standard talc, *p* < 0.0029 for modified talc), (Figure 3b).

These results suggest that modified talc application may be a significant alternative to standard talc application for the purpose of creating pleurodesis.

### 3.3. Histopathological Findings

**Figure 4:**
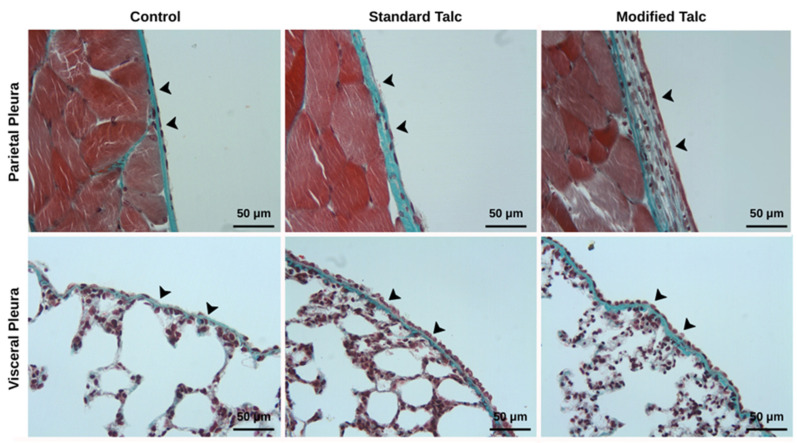
It is noteworthy that visceral and parietal pleural thickness (arrowheads) and collagen density increased in both the standard talc and modified talc groups.

An increase in both visceral and parietal pleural thickness was observed in the modified talc and standard talc groups compared to the control group (Figure 4). The increase in the modified talc group was statistically significant (*p* < 0.05), whereas the increase in the standard talc group did not reach statistical significance (Table 1 and Table 2). When comparing the two talc-treated groups, pleural thickness was greater in the modified talc group for both visceral and parietal pleura (*p* < 0.05, Figure 4, Table 1 and Table 2).

**Figure 5:**
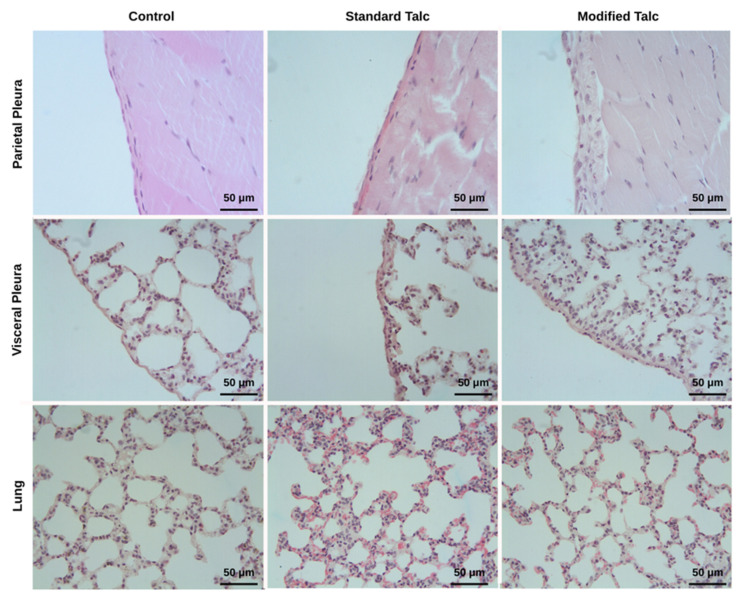
Granulation tissue increases in both pleura in the modified talc and standard talc groups. Mild damage is observed in the lung parenchyma in the standard talc and modified talc groups.

Granulation tissue formation was markedly increased in the modified talc group in both visceral and parietal pleura (Figure 5). This increase was significantly higher than that observed in the control and standard talc groups (*p* < 0.05). Although the standard talc group also showed an increase compared to the control, this difference was not statistically significant (Table 1 and Table 2).

Histopathological evaluation of the lung parenchyma revealed no significant differences between the groups. Mildly increased inflammatory infiltration and emphysematous areas were noted in both talc-treated groups (Figure 5), with changes slightly more pronounced in the standard talc group, although these differences were not statistically significant (Table 3).

Collagen density, assessed using Masson’s trichrome staining, was elevated in both the modified talc and standard talc groups compared to control (Figure 4). The increase in the modified talc group was significant relative to control (*p* < 0.05), whereas the increase in the standard talc group was not statistically significant. No significant difference was detected between the modified and standard talc groups (Table 1 and Table 2).

**Figure 6:**
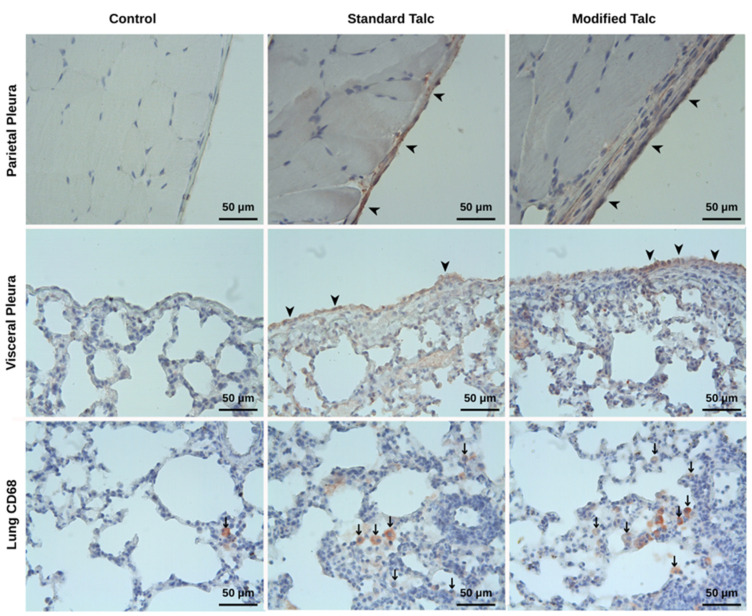
TGF-β immunoreactivity is observed to increase in the modified talc and standard talc groups (arrowheads). It is noteworthy that CD68(+) cells increase in the modified talc and standard talc groups (arrows).

TGF-β immunoreactivity was significantly increased in both the visceral and parietal pleura, particularly in the modified talc group, compared to the control group (*p* < 0.05). Although a slight increase was observed in the standard talc group, this change did not reach statistical significance (Figure 6, Table 1 and Table 2).

In the lung parenchyma, the number of CD68-positive cells was significantly higher in both the standard talc and modified talc groups compared to control (*p* < 0.05). Although the modified talc group exhibited a higher number of positive cells than the standard talc group, this difference was not statistically significant (Figure 6, Table 3).

## 4. Discussion

In this experimental study, the effect of a new talc formulation modified by adding antibacterial properties and combining lidocaine was evaluated in comparison with standard talc on pleurisy. Findings show that modified talc causes more pronounced inflammatory and fibrotic responses than standard talc when evaluated by histopathological parameters such as pleural thickness, granulation tissue formation, and collagen deposition. Furthermore, the significant increase in TGF-β expression and CD68-positive macrophage infiltration indicate that modified talc promotes both fibroblast activation and macrophage-mediated inflammatory responses. These results show that modified talc may provide effective pleurodesis by more effectively inducing inflammatory and fibrotic responses in both visceral pleura and parietal pleura tissues. However, biochemical analyses suggest that modified talc may induce a lower systemic inflammatory response compared to standard talc. This effect may be attributable to the anti-inflammatory properties of lidocaine in the modified talc structure or to the limited systemic permeation of the modified talc particles. Nevertheless, further studies are required to confirm this interpretation.

Recurrent pleural effusions arising from various etiologies, including malignant diseases, congestive heart failure, infections, and certain autoimmune disorders, are a significant clinical problem that adversely affects patients’ quality of life [17]. Similarly, recurrent pneumothorax is a condition requiring surgical intervention and has a high morbidity [18,19,20]. Pleurodesis is a treatment method that aims to permanently prevent air or fluid accumulation in the pleural space by creating adhesion between the visceral and parietal pleural layers [21]. Various sclerosing agents, such as bleomycin, doxycycline, doxorubicin, autologous blood, and tetracycline, have been applied for this purpose; however, standard talc remains the most commonly used and effective agent [22]. Although existing agents have the advantages of being easy to apply, low cost, effective, and safe, an ideal sclerosing agent that fully meets these criteria has not yet been developed.

Today, talc used for pleurodesis is one of the most preferred sclerosing agents due to its high inflammatory effect, which enables it to create permanent pleural adhesions, its high success rates, and its long-term effectiveness. However, it has safety limitations such as systemic inflammation and the risk of serious pulmonary complications depending on particle size; therefore, new forms of talc with enhanced biocompatibility at an appropriate particle size or new pleurodesis agents need to be developed to maintain its efficacy while reducing side effects [23,24].

Structural characterization of the modified talc was performed using Fourier Transform Infrared Spectroscopy (FTIR), while thermal properties were evaluated using thermogravimetric analysis (TGA) and differential scanning calorimetry (DSC). The antibacterial properties of the modified formulation were also assessed. Collectively, these structural improvements are expected to enhance both the efficacy and tolerability of modified talc compared to standard talc formulations. However, the long-term safety and efficacy of this new formulation, particularly in preventing recurrent malignant pleural effusions and recurrent pneumothoraces, have not yet been established. Therefore, large-scale, long-term clinical studies are required before modified talc can be recommended for routine clinical use. Nevertheless, this formulation has the potential to advance current pleurodesis practices and improve patient outcomes and quality of life.

In this study, the efficacy of a structurally modified talc formulation was compared with that of standard talc, currently the most widely used agent for pleurodesis. The effects of modified talc on histopathological parameters, including pleural thickness, granulation tissue formation, and inflammatory response, were evaluated in a rat model and compared with standard talc application. The results demonstrated that modified talc elicited a more pronounced fibrogenic and immune response than standard talc.

In our study, granulation tissue formation was significantly higher in the modified talc group in both the visceral and parietal pleura compared to the control and standard talc groups. This finding suggests that modified talc enhances the immune response and accelerates tissue repair processes. The observed increase in granulation tissue indicates that the early proliferative phase and regenerative mechanisms are actively engaged, consistent with findings reported in previous experimental studies [25,26]. Although an increase in granulation tissue was also noted in the standard talc group, this increase did not reach statistical significance, suggesting that classical talc may trigger an inflammatory response to a lesser extent. Modified talc administration caused a significant increase in both visceral and parietal pleural thickness compared to the control and standard talc groups, consistent with the increase in granulation tissue. The marked increase in parietal pleural thickness particularly indicates that modified talc induces a stronger local tissue response and fibrogenic process than standard talc. This observation aligns with previous studies demonstrating the capacity of talc to induce pleural thickening and fibrosis [27].

Collagen deposition, assessed by Masson’s trichrome staining, was significantly higher in the modified talc group compared to the control, suggesting that modified talc promotes fibroblast activation and subsequent collagen synthesis. In the fibrotic process, characterized by fibroblast activation and extracellular matrix accumulation and associated with increased profibrotic cytokines such as TGF-β, the effect of modified talc was particularly pronounced. Although collagen deposition was also observed in the standard talc group, this increase was not statistically significant. This indicates that talc itself can trigger the fibrotic process, while the structural modification enhances this effect. It has been reported that TGF-β promotes collagen synthesis and suppresses IL-8 production in pleural mesothelial cells, which may contribute to the effects observed with modified talc. Furthermore, the absence of a significant difference between the modified and standard talc groups suggests that both formulations can induce similar fibrotic responses; however, the modified form may exert a stronger effect [28]. Taken together, these findings indicate that modified talc produces a more pronounced inflammatory and fibrotic response than standard talc and may thus be more effective in achieving pleural adhesion. Nonetheless, the impact of this robust tissue response on long-term pleural integrity and respiratory function warrants further investigation.

In this study, the immunological and histopathological effects of intrapleural administration of standard and modified talc for pleurodesis in rats were investigated. Modified talc was shown to produce significantly higher TGF-β immunoreactivity in both the visceral and parietal pleura compared to the control group. Although a slight increase in TGF-β was observed in the standard talc group, this increase was not statistically significant. TGF-β is an important cytokine in the fibrogenesis process that regulates the fibrotic response by promoting fibroblast proliferation and extracellular matrix accumulation [29,30]. In this context, the elevated TGF-β observed with modified talc indicates that it causes a stronger fibrotic response than that induced by standard talc. Additionally, the number of CD68-positive macrophages in lung tissue increased significantly in both the standard and modified talc groups. CD68 is a macrophage marker and is commonly used to assess the degree of inflammatory response [31]. Although the modified talc group had a higher number of CD68-positive cells than the standard talc group, this difference was not statistically significant. Nevertheless, the significant increase in both groups compared to the control supports the notion that talc-based pleural agents enhance inflammatory cell infiltration and that macrophage activation contributes substantially to pleural fibrosis [32]. These findings indicate that intrapleural modified talc application not only stimulates the fibrotic process but also increases macrophage-mediated inflammation. Considering the concomitant increase in TGF-β and CD68 expression, it can be inferred that modified talc initiates a more intense and sustained inflammatory–fibrotic response on the pleural surface. Clinical studies report that inflammation and subsequent fibrosis are the main determinants of the effectiveness of pleural sclerosing agents. Accordingly, our findings suggest that modified talc may be a more potent sclerosing agent than standard talc and may be particularly advantageous in clinical scenarios where durable pleural adhesion is desired.

Histopathological examination of the lung parenchyma revealed mild inflammatory cell infiltration and emphysema-like changes in both groups treated with modified talc and standard talc. The absence of a significant difference between the groups indicates that the effects of these agents are largely localized to the pleura and have minimal systemic or parenchymal side effects; this finding supports the safe use of these agents. Previous studies similarly report that intrapleural talc may induce only minor histopathological changes in the lung parenchyma [33]. It has also been noted that talc preparations with smaller particle sizes can cause more widespread or pronounced pulmonary responses; therefore, particle size should be considered in pleurodesis procedures using talc. Overall, modified talc elicited more pronounced fibrogenic and immune responses than standard talc, as evidenced by increased granulation tissue formation, collagen deposition, and pleural thickening. These findings suggest that modified talc may enhance the likelihood of achieving effective pleural adhesion, potentially offering advantages in clinical contexts requiring tissue repair. However, its long-term effects on lung parenchyma and overall safety profile remain to be fully determined. Therefore, more comprehensive clinical studies are needed to thoroughly evaluate the efficacy and safety of modified talc.

Although lidocaine was incorporated into the modified talc formulation to reduce procedural discomfort, the absence of a validated pain assessment method represents a limitation of the present study. Future experimental studies should incorporate standardized pain scoring systems to objectively evaluate the potential analgesic benefit of lidocaine-enriched pleurodesis agents.

## 5. Conclusions

In this study, we demonstrated that a surface-modified talc formulation, enriched with antibacterial properties and supplemented with lidocaine, induced stronger inflammatory and fibrotic responses in rats compared to standard talc in terms of pleurodesis efficacy. The modified talc structure significantly increased pleural thickness and promoted granulation tissue formation and collagen deposition. No significant damage was observed in the lung tissue after pleurodesis, indicating that the effects of the application were localized and well tolerated. In conclusion, modified talc pleural adhesion may be a potential alternative to standard talc in creating pleural adhesion for the treatment of recurrent pleural effusion and pneumothorax by enhancing the effectiveness of pleurodesis. However, these findings were obtained from an experimental animal model and should be confirmed by clinical studies in humans. Furthermore, the potential systemic effects and long-term safety of modified talc should be comprehensively evaluated in future studies.

## Figures and Tables

**Table 1:** Histological and immunohistochemical findings in the visceral pleura.

Groups	Control	Standard Talc	Modified Talc
Thickness (μm)	9.02 (6.99–10.00)	9.35 (8.34–13.41)	12.47 (10.12–16.1) ^a,b^
Granulation	0 (0–1)	1 (0–1)	2 (1–3) ^a,b^
Collagen Density	0 (0–0)	0 (0–1)	0.50 (0–1) ^a^
TGF-β Immunoreactivity	0 (0–0)	0 (0–2)	1.50 (0–4) ^a^

Data are presented as median (min–max) values. ^a^ Increase compared to the control group (*p* < 0.05); ^b^ Increase compared to the standard talc group (*p* < 0.05).

**Table 2:** Histological and immunohistochemical findings in the parietal pleura.

Groups	Control	Standard Talc	Modified Talc
Thickness (μm)	9.49 (7.26–11.67)	13.52 (9.51–14.86)	17.82 (15.61–25.55) ^a,b^
Granulation	0 (0–1)	1 (0–2)	2 (1–3) ^a,b^
Collagen Density	0 (0–0)	0 (0–1)	1 (0–2) ^a^
TGF-β Immunoreactivity	0 (0–0)	0 (0–2)	1 (0–6) ^a^

Data are presented as median (min–max) values. ^a^ Increase compared to the control group (*p* < 0.05)^; b^ Increase compared to the standard talc group (*p* < 0.05).

**Table 3:** CD68-positive cell count and histopathological score in lung tissue.

Groups	Control	Standard Talc	Modified Talc
CD68 (+) Cell Count	3 (3–14)	16 (12–22) ^a^	21.50 (12–29) ^a^
Histopathological Score	1 (0–2)	1 (1–2)	1 (0–2)

Data are presented as median (min–max) values. ^a^ Increase compared to the control group (*p* < 0.05).

## Data Availability

All data generated or analyzed during this study are included in this published article.
